# A case of primary bacteraemia caused by *Salmonella enterica* serovar Corvallis in an immunocompetent adult after travel to Southeast Asia

**DOI:** 10.1099/acmi.0.000009

**Published:** 2019-03-25

**Authors:** Sho Nakakubo, Kentaro Nagaoka, Masaru Suzuki, Satoshi Konno, Yasushi Shibue, Tetsuya Ikeda, Masaharu Nishimura

**Affiliations:** 1 First Department of Internal Medicine, Hokkaido University Hospital, Hokkaido, Japan; 2 Division of Infectious Disease, Tokyo Takanawa Hospital, Tokyo, Japan; 3 Hokkaido Instititute of Public Health, Hokkaido, Japan

**Keywords:** non-typhoidal *Salmonella*, bacteraemia, *Salmonella enterica* serovar Corvallis, tropical infection, traveller

## Abstract

**Introduction:**

Non-typhoidal *Salmonella* (NTS) that typically causes diarrhoeal disease in humans has a dramatically more severe and more invasive presentation than typhoid fever in immunocompromised adults. However, the incidence and significance of NTS primary bacteraemia in immunocompetent adults have been unclear.

**Case presentation:**

A 24-year-old man presented to our hospital with a high fever 14 days after travelling to Vietnam and Cambodia for 14 days. His past medical history, family history and social history were unremarkable, except for his dietary intake history during his stay in Southeast Asia. He did not have any abdominal pain, diarrhoea, enterocolitis, arthritis, or abscesses, as determined by multiple examinations, which included computed tomography. The initial blood cultures identified the presence of Gram-negative bacilli, which were finally identified as the *Salmonella enterica* subspecies serovar Corvallis. Thus, *S. enterica* serovar Corvallis was the most likely primary bacteria in this patient. Since domestic outbreaks of NTS infections are extremely rare, our case patient was diagnosed with travel-related bacteraemia. The patient had an uneventful recovery after antibiotic administration.

**Conclusion:**

We report a rare case of bacteraemia caused by *S. enterica* serovar Corvallis in an immunocompetent adult after travelling through Vietnam and Cambodia. From the experience of our case, we suggest that more caution is necessary when diagnosing the unique clinical features of travel-related NTS infections.

## Introduction

*Salmonellae* are ubiquitous Gram-negative bacilli that cause foodborne, zoonotic and travel-related infections. Among *Salmonella* species, *Salmonella enterica* serovar Typhi and Paratyphi are human-restricted. They cause a primary bacteraemia that is known as typhoid fever, a well-known traveller’s fever that occurs in endemic regions. The non-typhoidal *Salmonella* (NTS) that typically cause diarrhoeal disease in humans have a diverse range of hosts, including many vertebrate animals that are used for human consumption [1]. In contrast to typhoid fever, NTS has a dramatically more severe and more invasive presentation than typhoid fever in immunocompromised adults, particularly in patients with HIV infections and/or malignancies [2]. However, the incidence and significance of NTS primary bacteraemia in immunocompetent adults have been unclear. Here, we report a case of primary bacteraemia that was caused by the NTS *Salmonella enterica* serovar Corvallis in a previously healthy adult after travel throughout Southeast Asia.

## Case Report

A 24-year-old native Japanese man presented to our hospital after developing high-grade fever. He had travelled to Vietnam and Cambodia for 14 days. After leaving Japan, he visited the beach in Halong Bay, the forest region near the border of China, and Siem Reap in Cambodia. During his stay, he had participated in forest trekking tourism and had stayed in a youth hostel. For dietary intake, he ate home-cooked food and drank beverages with ice, in a similar manner to local people. Fourteen days after he returned to Japan, he developed a mild headache, chills and malaise, and 2 days later, he developed a high fever with chills and rigors. He did not have any abdominal pain or diarrhoea. His past medical history, family history and social history were unremarkable. On examination, his temperature was 39 ℃, his blood pressure was 128/68 mmHg, his pulse was 101 beats per minute, his respiratory rate was 18 breaths per minute and his oxygen saturation was 98 % with room air. He did not present a rash, lymph node swelling, abdominal tenderness, or hepatosplenomegaly. His laboratory examination demonstrated elevated serum C-reactive protein (5.1 mg dl^−1^; normal range, 0–0.3 mg dl^−1^). The blood cell counts and the other biochemical tests were normal (WBC 6900 µl^−1^, AST 20 U l^−1^, ALT 19 U l^−1^). The HIV test result was negative, while contrast-enhanced computed tomography (CT) revealed ileocecal lymphadenopathy. We did not detect enterocolitis, arthritis, or abscesses. We also did not detect an aneurysm or abscess via brain magnetic resonance imaging (MRI).

Based on his travel history and clinical findings, we suspected primary bacteraemia from typhoid fever. He was admitted to our hospital for observation and treated with antibiotics. On the day of admission, ceftriaxone (2 g/day) and minocycline (200 mg/day) were administered. On hospital day two, the initial blood cultures yielded Gram-negative bacilli, which were identified as *Salmonella* by matrix-assisted laser desorption/ionization time-of-flight mass spectrometry. The bacterial subspecies could not be identified by biochemical testing at our hospital. Antibiotic susceptibility testing revealed that the organism was not resistant to the following antibiotics: ampicillin, piperacillin, cefotiam, cefotaxime, ceftizoxime, ceftazidime, cefepime, imipenem, meropenem, azythromycin, levofloxacin, ciprofloxacin and fosfomycin. Only minomycin had an MIC_90_ value of >8 µg ml^−1^.

After 1 day of ceftriaxone and minocycline, his fever, malaise and headache remitted. We discontinued minocycline, and ceftriaxone was continued for 14 days. The patient was discharged on day 10 and had an uneventful recovery with no recurrence of fever.

Further testing using commercial O and H antisera (Denka Seiken Co. Ltd, Tokyo, Japan) according to the *Antigenic Formulae of the Salmonella Serovars* (9th ed.) [3] identified the organism as *Salmonella enterica* serovar Corvallis (at Hokkaido Institute of Public Health, Sapporo, Japan). Thus, *S. enterica* serovar Corvallis was likely to have caused the primary bacteraemia in this patient, as described.

## Discussion

*S. enterica* serovar Corvallis has been reported worldwide as a bacterium that causes human gastroenteritis, since its initial isolation from poultry in 1949 [4]. Antibiotic-resistant *S. enterica* serovar Corvallis has been reported as an emerging concern. Surveillance programmes that have been conducted in Thailand, Bulgaria and Denmark have revealed that *S. enterica* serovar Corvallis contains extended-spectrum beta-lactamase (ESBL) resistant genes [5]. In Japan, a strain of *S. enterica* serovar Corvallis containing the *qnr* gene, the quinolone resistance-determining region, was isolated from patients with overseas travellers’ diarrhoea [6]. In Germany, a strain containing New Delhi metallo-beta-lactamase (NDM) was isolated from a wild bird (black kite), which indicated that NDM-producing *Salmonella* could be spread via migratory birds from an endemic location [7]. *S. enterica* serovar Corvallis has been detected in humans and food products worldwide, including in the United States, Southeast Asia, North Africa and Nigeria [8]. In Cambodia, the examination of 152 poultry carcasses from retail outlets in Phnom Penh revealed that 88.2 % of poultry samples were highly contaminated with NTS; *S. enterica* serovar Corvallis constituted 5.9 % of the total *Salmonella* isolates [9]. In Japan, *S. enterica* serovar Corvallis was reported to constitute 15.1 % of the NTS isolated from 106 (0.032 %) out of 331 644 faecal samples from food-handlers [10]. Although there has been no report of an infection caused by multidrug-resistant *S. enterica* serovar Corvallis, the potential for this organism to spread warrants judicious surveillance.

Our patient seemed to be infected by *S. enterica* serovar Corvallis during a travel period, since he had no suspicious food intake during his time in Japan. Fortunately, the isolated strain in this case was drug-susceptible. The patient recovered soon after receiving antibiotics, and the treatment duration of 14 days was sufficient, which corresponded to that required for general bacteraemia. However, primary bacteraemia due to *S. enterica* serovar Corvallis in an immunocompetent patient is extremely rare, and seldom occurs in domestic situations, and this could be the first documented case of travel-related *S. enterica* serovar Corvallis primary bacteraemia in Japan.

Accommodations, travel characteristics and dietary hygiene are known to be important factors in the contraction of travellers’ diarrhoea, since these factors can contribute to the ingestion of potentially contaminated food [11]. Koch *et al*. reported that travel-related NTS bacteraemia was more frequent than domestically acquired NTS bacteraemia in healthy adults in a Danish population-based cohort study [12]. In their study, travel-related NTS bacteraemia affected 71.1 % of healthy adults without comorbidities and presented a lower risk of death after 30–90 days than domestically acquired bacteraemia. Until now, primary bacteraemia due to NTS in domestic situations has been associated with high mortality and underlying malignancies and immunosuppression [13]. Although Choleraesuis, Virchow and Panamas are known as invasive *Salmonella* serovars [13, 14], few studies have investigated invasive infections caused by *S. enterica* serovar Corvallis.

In our case, primary NTS bacteraemia due to *S. enterica* serovar Corvallis occurred in the immunocompetent patient after travel throughout Southeast Asia. As to why bacteraemia occurred without gastroenteritis, not only an intake of a relatively large bacterial load via contaminated food, but also strain-dependent virulence, might be responsible for the pathogenesis of primary bacteraemia. With respect to the mechanism of NTS infection, it is known that NTS invades into the epithelial cells of the gut mucosa, which enables the avoidance of neutrophil-mediated killing [15]. *Salmonellae* invasion and intracellular replication within host cells results in a range of diseases, including bacteraemia. Given these pathologies, the primary bacteraemia in our case might be partly due to the stronger invasion of the bacteria, which is affected by regional characteristics. Although the detailed mechanism is unclear, from the experience of our case, we suggest the possibility that more virulent strains of *S. enterica* serovar Corvallis, which express stronger invasion within host cells, might exist in some locations. Given this and the emerging drug resistance of this species, more caution is necessary regarding the spread of *S. enterica* serovar Corvallis via travellers.

In conclusion, we report a rare case of primary bacteraemia that was caused by *S. enterica* serovar Corvallis in an immunocompetent adult who travelled through Vietnam and Cambodia. From the experience of our case, we suggest that more caution is necessary with respect to the detection of the unique clinical features of travel-related NTS infections.

## References

[1] Fierer J, Swancutt M (2000). Non-typhoid *Salmonella*: a review. Curr Clin Top Infect Dis.

[2] Shimoni Z, Pitlik S, Leibovici L, Samra Z, Konigsberger H (1999). Nontyphoid *Salmonella* bacteremia: age-related differences in clinical presentation, bacteriology, and outcome. Clin Infect Dis.

[3] Grimont PAD, Weill FX, Popoff MY, Le Minor L (2007). Antigenic Formulas Of the Salmonella Serovars.

[4] Edwards PR, Hermann GJ (1949). Two new Salmonella types; *Salmonella* corvallis and *Salmonella* colorado. J Bacteriol.

[5] Archambault M, Petrov P, Hendriksen RS, Asseva G, Bangtrakulnonth A (2006). Molecular characterization and occurrence of extended-spectrum beta-lactamase resistance genes among *Salmonella*
*enterica* serovar Corvallis from Thailand, Bulgaria, and Denmark. Microb Drug Resist.

[6] Taguchi M, Kawahara R, Seto K, Inoue K, Hayashi A (2009). Plasmid-mediated quinolone resistance in *Salmonella* isolated from patients with overseas travelers' diarrhea in Japan. Jpn J Infect Dis.

[7] Fischer J, Schmoger S, Jahn S, Helmuth R, Guerra B (2013). NDM-1 carbapenemase-producing *Salmonella enterica* subsp. enterica serovar Corvallis isolated from a wild bird in Germany. J Antimicrob Chemother.

[8] Hendriksen RS, Vieira AR, Karlsmose S, Lo Fo Wong DM, Jensen AB (2011). Global monitoring of Salmonella serovar distribution from the World Health organization global foodborne infections network country data bank: results of quality assured laboratories from 2001 to 2007. Foodborne Pathog Dis.

[9] Lay KS, Vuthy Y, Song P, Phol K, Sarthou JL (2011). Prevalence, numbers and antimicrobial susceptibilities of *Salmonella* serovars and *Campylobacter* spp. in retail poultry in Phnom Penh, Cambodia. J Vet Med Sci.

[10] Murakami K, Ishihara T, Horikawa K, Oda T (2007). Features of *Salmonella* serovars among food handlers in Kyushu, Japan. New Microbiol.

[11] Kollaritsch H (1989). Traveller's diarrhea among Austrian tourists in warm climate countries: I. Epidemiology. Eur J Epidemiol.

[12] Koch K, Kristensen B, Holt HM, Ethelberg S, Mølbak K (2011). International travel and the risk of hospitalization with non-typhoidal *Salmonella* bacteremia. A Danish population-based cohort study, 1999–2008. BMC Infect Dis.

[13] Gordon MA (2008). *Salmonella* infections in immunocompromised adults. J Infect.

[14] Jones TF, Ingram LA, Cieslak PR, Vugia DJ, Tobin-D'Angelo M (2008). Salmonellosis outcomes differ substantially by serotype. J Infect Dis.

[15] LaRock DL, Chaudhary A, Miller SI (2015). *Salmonellae* interactions with host processes. Nat Rev Microbiol.

